# Correspondence

**Published:** 1882-04

**Authors:** 


### CORRESPONDENCE.

EDITOR JOURNAL OF COMPARATIVE MEDICINE AND SURGERY:—

A few years ago I turned a fat cow into a small yard in which had been a bed of onions, in the harvesting of which many imperfect ones had been left. After remaining in the yard about seven hours she was killed. The muscle was found untainted by the onions she had eaten; the fat emitted a very appreciable odor when cooked; the marrow when cooked emitted so strong and offensive an odor as to make it very unpleasant to remain in the kitchen. The three experiments were tried the same day the cow was killed. May not the comparative physiologist learn something from this as to the assimilation of food and the rapidity of the deposits from the blood in the various parts of the system? May not this suggest further experiments in the same direction by more competent observers?

J. D. CATON.

OTTAWA, ILL.

